# Cellular mechanisms of phase maintenance in the pyloric motif

**DOI:** 10.1186/1471-2202-14-S1-P76

**Published:** 2013-07-08

**Authors:** Gabrielle E O'Brien, William H Barnett, Gennady S Cymbalyuk

**Affiliations:** 1 Mathematics, Agnes Scott College, Decatur, GA, 30030, USA; 2 Neuroscience Institute, Georgia State University, Atlanta, GA, 30303, USA

## 

Many biological motor patterns such as leech and lamprey swimming, ventilation in crabs and food particle filtration in crustaceans are maintained in a range of frequencies. This phenomenon is well-studied in the crustacean pyloric network, a central pattern generator (CPG) which produces a distinct bursting pattern with three-to-fivefold changes in period [1-4]. The flexibility of the network output requires neurons to adjust their temporal characteristics, such as burst duration, interburst interval and time to firing from synaptic input, in order to maintain their phase in the network pattern as the period changes. Recently, it has been shown that the coordinated neuromodulation of currents in pyloric neurons is a cellular mechanism sufficient to support phase maintenance [3,4].

In the model neuron reported here, coordinated variation of the voltages of half-activation for the potassium (θ_K2_) and hyperpolarization-activated (θ_h_) currents provides a family of dynamical mechanisms for fine control of burst duration, interburst interval and latency to spiking. The mechanisms are determined by a global codimension-2 bifurcation, the Cornerstone bifurcation with bifurcation parameters θ_K2 _and θ_h._. In Mechanism 1, the burst duration of a bursting neuron grows arbitrarily large as θ_K2 _approaches a blue sky catastrophe, and the interburst interval grows arbitrarily large as θ_h _approaches a SNIC bifurcation. We found level sets of duty cycle using an analytical approximation imposed by the Cornerstone bifurcation. These level sets describe coordination of the parameters that could be realized through neuromodulation. In Mechanism 2, a silent neuron exhibits a single burst in response to synaptic inhibitory input; the duration of the burst is determined by θ_K2_. In Mechanism 3, a spiking neuron exhibits a pause before firing in response to synaptic inhibitory input; the duration of the pause is determined by θ_h_.

**Figure 1:**
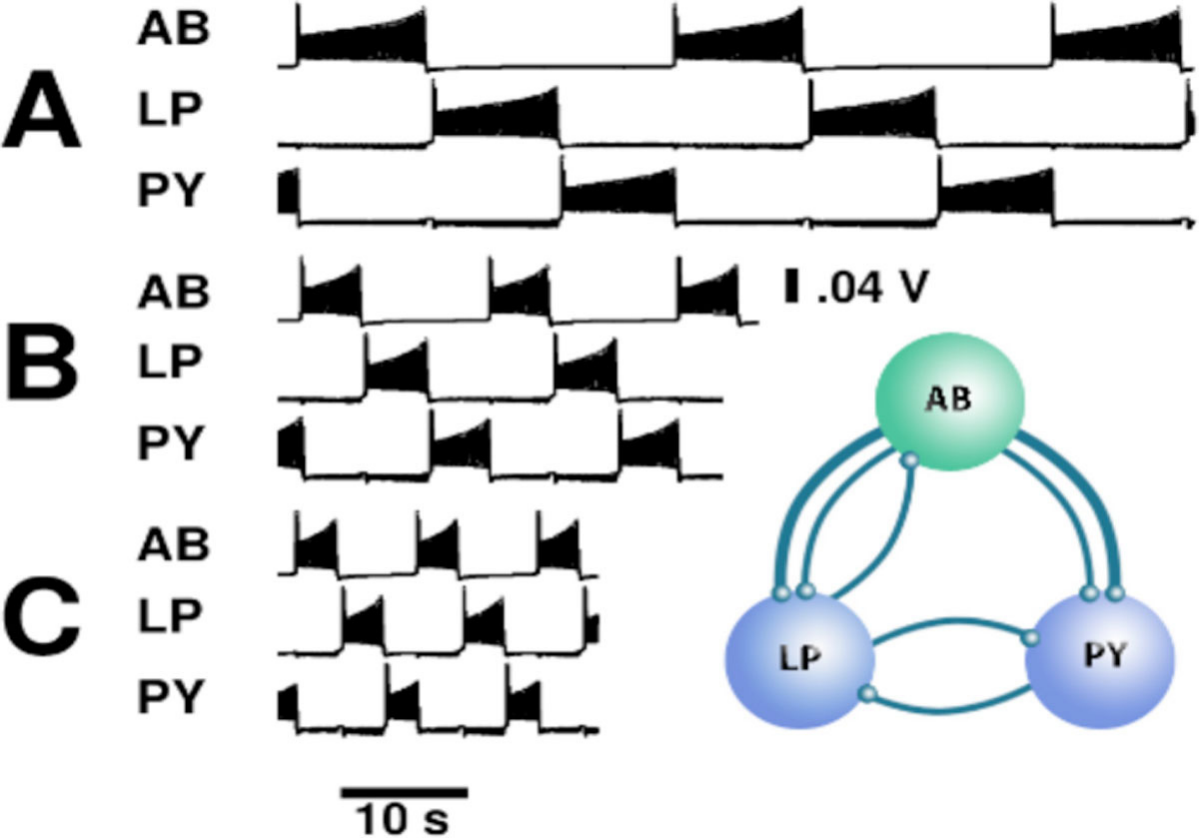
**Phase maintenance in a pyloric motif with a bursting driver and two silent follower neurons at 10, 16 and 32 second periods**. Mechanism 1 controls the burst duration and interburst interval of the pacemaker neuron. Mechanism 2 controls the burst duration of the two follower neurons. The pattern was maintained with fourfold variation in period.

When applied to neurons connected in a pyloric motif, the family of mechanisms allows the neurons to maintain phase with one another as period grows up to fivefold. We consider three models: all three neurons are endogenously bursting (Mechanism 1), a pacemaker neuron is bursting and the follower neurons are silent (Mechanisms 1 and 2, Figure 1), and a pacemaker neuron is bursting and the follower neurons are spiking (Mechanisms 1 and 3). In each model, the pacemaker neuron maintains duty cycle with increasing period and the follower neuron's parameters are coordinated to match. These mechanisms are generic, cellular in essence, and not sensitive to synaptic parameters.
